# Identification of Highly Repetitive Enhancers with Long-range Regulation Potential in Barley via STARR-seq

**DOI:** 10.1093/gpbjnl/qzae012

**Published:** 2024-02-21

**Authors:** Wanlin Zhou, Haoran Shi, Zhiqiang Wang, Yuxin Huang, Lin Ni, Xudong Chen, Yan Liu, Haojie Li, Caixia Li, Yaxi Liu

**Affiliations:** State Key Laboratory of Crop Gene Exploration and Utilization in Southwest China, Sichuan Agricultural University, Chengdu 611130, China; Triticeae Research Institute, Sichuan Agricultural University, Chengdu 611130, China; Triticeae Research Institute, Sichuan Agricultural University, Chengdu 611130, China; Chengdu Academy of Agricultural and Forestry Sciences, Chengdu 611130, China; State Key Laboratory of Crop Gene Exploration and Utilization in Southwest China, Sichuan Agricultural University, Chengdu 611130, China; Triticeae Research Institute, Sichuan Agricultural University, Chengdu 611130, China; Triticeae Research Institute, Sichuan Agricultural University, Chengdu 611130, China; Triticeae Research Institute, Sichuan Agricultural University, Chengdu 611130, China; Triticeae Research Institute, Sichuan Agricultural University, Chengdu 611130, China; Triticeae Research Institute, Sichuan Agricultural University, Chengdu 611130, China; Triticeae Research Institute, Sichuan Agricultural University, Chengdu 611130, China; Triticeae Research Institute, Sichuan Agricultural University, Chengdu 611130, China; State Key Laboratory of Crop Gene Exploration and Utilization in Southwest China, Sichuan Agricultural University, Chengdu 611130, China; Triticeae Research Institute, Sichuan Agricultural University, Chengdu 611130, China

**Keywords:** Barley, Enhancer, Transposable element, Gene expression, Repetitive sequence

## Abstract

Enhancers are DNA sequences that can strengthen transcription initiation. However, the global identification of plant enhancers is complicated due to uncertainty in the distance and orientation of enhancers, especially in species with large genomes. In this study, we performed self-transcribing active regulatory region sequencing (STARR-seq) for the first time to identify enhancers across the barley genome. A total of 7323 enhancers were successfully identified, and among 45 randomly selected enhancers, over 75% were effective as validated by a dual-luciferase reporter assay system in the lower epidermis of tobacco leaves. Interestingly, up to 53.5% of the barley enhancers were repetitive sequences, especially transposable elements (TEs), thus reinforcing the vital role of repetitive enhancers in gene expression. Both the common active mark H3K4me3 and repressive mark H3K27me3 were abundant among the barley STARR-seq enhancers. In addition, the functional range of barley STARR-seq enhancers seemed much broader than that of rice or maize and extended to ±100 kb of the gene body, and this finding was consistent with the high expression levels of genes in the genome. This study specifically depicts the unique features of barley enhancers and provides available barley enhancers for further utilization.

## Introduction

Enhancers are among the most important *cis*-regulatory modules and help plants coordinate both developmental and environmental responses [1]. These common elements act in upstream or downstream of genes, and they are always located in distal promoter regions [2]. In recent years, many enhancers have been found to regulate developmental or tolerance processes, such as the inflorescence architecture, phosphate homeostasis, shoot regeneration, floral transition, and salinity stress tolerance [3–7]; thus, these elements show good potential in regulating various functional genes. However, enhancers are difficult to identify because their position and orientation are uncertain.

Multiple sequencing methods have been used to identify enhancers in plants. DNA and chromatin features have been used to predict enhancer candidates in both animal and plant genomes. DNase I hypersensitive sites (DHSs) are commonly associated with enhancers, silencers, promoters, insulators, and locus control regions [8]. However, the follow-up standard Southern blot approach is a complicated, time-consuming, and inaccurate process and thus represents a huge constraint in enhancer exploration [9]. To address this issue, massively parallel signature sequencing (MPSS) was combined with DHS mapping to promote genome-wide enhancer identification [9]. DHS data were recently obtained for species with relatively small genomes, such as *Arabidopsis*, maize, tomato, sorghum, foxtail millet, and rice [10–12]. The assay for transposase-accessible chromatin with sequencing (ATAC-seq) method was recently introduced for the discovery of *cis*-regulatory regions in plants [13]. However, the regulatory units identified by ATAC-seq are tissue-specific, thereby masking partial enhancers. DNA methylation and histone modification are considered important marks for identifying *cis*-regulatory units of grass genomes [14,15]. In maize, DNA methylation has been successfully used to explore unmethylated regions (UMRs) that carry potentially active promoters and *cis*-regulatory units [15]. Thus, chromatin immunoprecipitation sequencing (ChIP-seq) technology has been utilized to discover enhancer sites with symbolic H3K4me1 and H3K27ac modifications in large quantities, thus offering the possibility of identifying super enhancers (SEs) [16–18]. Although the aforementioned methods can identify valid enhancers, only a limited number of enhancers have been found by DNA and RNA labels, and the current protocols are too expensive and labor-intensive to apply to plant genomes.

Self-transcribing active regulatory region sequencing (STARR-seq), clustered regularly interspaced short palindromic repeats (CRISPR)-based approaches, and massively parallel reporter assay (MPRA) are the three main methods for combining genomic identification with the functional utilization of enhancers. MPRA tests thousands of sequences and nucleotide variants to reveal potential enhancer activities and is widely utilized in human and mammalian cells [19]; CRISPR-based enhancer identification mainly demonstrates intrinsic transcriptional and epigenetic reciprocity between human enhancers and promoters via dCas9-based activators [20]; and STARR-seq is widely used to directly and quantitatively assess enhancer activity for millions of candidates from arbitrary sources of DNA in both mammalian and non-mammalian genomes [21,22]. Compared to other sequencing methods, STARR-seq can quantify enhancer strength in complex candidate libraries [23]. All enhancers, whether active, chromatin-masked, or dormant, can be identified through STARR-seq [24]. Enhancers in the genomes of *Drosophila*, humans, and mice have been successfully obtained based on the library construction protocol [21,24–26], and this method can provide a considerable number of candidates for further functional studies in animals. With the advancement of library construction by protoplast transfection, quantitative enhancers in rice and maize have also been discovered [27,28], and modifications of this method allow enhancers to be identified by STARR-seq and validated in transient tobacco systems [29]. Moreover, a novel STARR-seq variant that includes unique molecular identifiers (UMIs), UMI-STARR-seq, is developed to accurately count reporter messenger RNAs (mRNAs) in low-complexity STARR-seq libraries, thus increasing the accuracy of global enhancer identification [30].

Although *cis*-regulation by enhancers is vital, few enhancers have been validated in Triticeae crops, such as wheat and barley. In 1987, a 268-bp enhancer-like sequence was discovered in the 5′-proximal region of the wheat chlorophyll a/b-binding-1 (*cab-1*) gene [31]; and in 1994, an enhancer/silencer sequence that directs aleurone-specific expression of a barley chitinase (*Chi26*) gene was identified [32]. Approximately three decades passed, only a few enhancers have been identified in barley and wheat due to the genome complexity and bulkiness. In this study, enhancers across the barley genome were identified by STARR-seq for the first time, and their strengths were predicted and quantified. The specific location distribution, sequence signature, and long-range regulation potential of barley enhancers, which differed from those of rice or maize, were also revealed.

## Results

### A total of 7323 enhancers were identified in barley using STARR-seq

After STARR-seq, two replicates were merged to identify possible enhancers. In total, 398,293,684 reads were acquired in the input plasmid libraries, and the fragments had a median length of 518 bp; and 512,678,889 reads were obtained in the complementary DNA (cDNA) libraries, and the fragments had a median length of 535 bp. After eliminating duplicates, 98,972,877 reads were reserved in the input plasmid libraries and 111,427,056 reads were reserved in the cDNA libraries.

**Figure 1:**
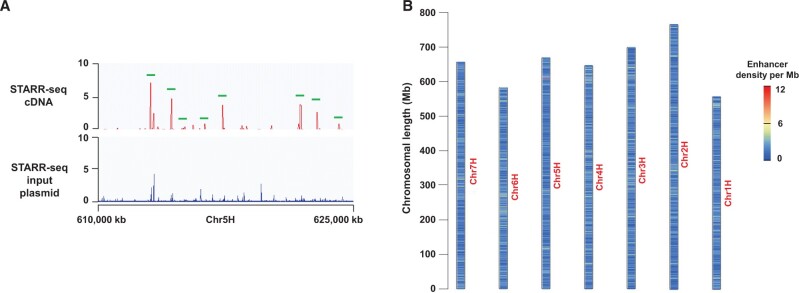
Genomic discovery of barley STARR-seq enhancers **A**. Merged STARR-seq input plasmid (blue) and cDNA (red) reads at representative genomic regions on Chr5H. Green boxes represent potential enhancer peaks. **B**. Distribution of barley STARR-seq enhancers across 7 chromosomes. STARR-seq, self-transcribing active regulatory region sequencing; Chr, chromosome; cDNA, complementary DNA.

In total, 7323 enriched peaks were successfully identified and used for follow-up analysis (**Figure 1**A; Table S1). Analysis of chromosomal locations indicated that 7323 barley STARR-seq enhancers were randomly located on 7 chromosomes without obvious location preferences, indicating that our identification is genome-wide (Figure 1B).

### Few STARR-seq enhancers overlapped with ATAC-seq peaks in barley

Compared with previously published barley ATAC-seq peaks, our STARR-seq enhancers were more balanced in terms of distribution and density (Figure 1B, Figure S1). Interestingly, STARR-seq enhancers in rice were more balanced than ATAC-seq enhancers as well [27,33] (Figure S2). ATAC-seq peaks in rice, maize, and barley [33] were analyzed and found to be enriched at the ends of each chromosome, which is possibly due to the bias in chromosome cleavage via transposase Tn5 (Figures S1–S3). The difference in distribution features further revealed that the STARR-seq method was less affected by chromosomal location or orientation. Meanwhile, barley ATAC-seq peaks were identified through the same reference genome and parameters and compared with our STARR-seq peaks. Surprisingly, only a few STARR-seq enhancers (∼ 0.9%) overlapped with ATAC-seq peaks in barley (Table S1). Similar to the poor intersection (∼ 8.7%) observed between STARR-seq and DHS-seq enhancers and the poor intersection (∼ 4.0%) between STARR-seq and ATAC-seq enhancers in rice, the low overlap proportion among STARR-seq, ATAC-seq, and DHS-seq in plants was likely caused by different library construction principles and sampling statuses [27,33]. Accessible chromatin regions (ACRs), for example, included much more than enhancers and had time, condition, even cell specificity in grass genomes [15].

### Low proportion of barley enhancers regulated adjacent target genes

**Figure 2:**
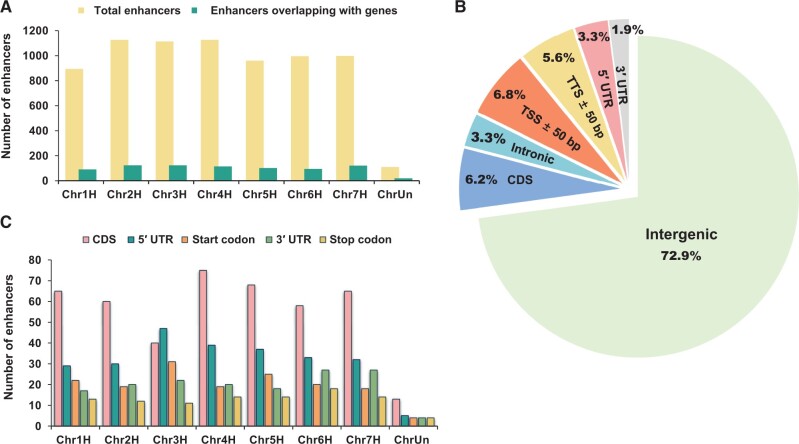
Position distribution characteristics of barley STARR-seq enhancers **A**. Number of total enhancers and enhancers overlapping with genes in each chromosome. **B**. Proportion of enhancers overlapping with or without various gene elements. **C**. Number of enhancers overlapping with CDS, 5′ UTR, start codon, 3′ UTR, and stop codon regions. TSS, transcription start site; TTS, transcription termination site; CDS, coding sequence; 5′ UTR, 5′ untranslated region; 3′UTR, 3′ untranslated region; ChrUn, unknown chromosome.

Among the total barley STARR-seq enhancers, only 9%–11% in each chromosome directly overlapped with coding genes (**Figure 2**A). Furthermore, we searched for genes 10 kb upstream or downstream of the 7323 enhancers and found that a low proportion of enhancers (∼ 2735; ∼ 37%) had potential target genes at a distance of 10 kb, indicating that only a minority of genes in barley are regulated by immediately adjacent enhancers. However, in the rice genome, proximal regulation (±5 kb) by enhancers may be more common because a proportion of over 70% was observed [27].

### Barley enhancers were enriched in intergenic regions

To determine the location preference of barley STARR-seq enhancers, all chromosomal locations of various gene motifs in barley, including intergenic regions, intronic regions, transcription start sites (TSSs), 5′ untranslated regions (5′ UTRs), coding sequences (CDSs), 3′ untranslated regions (3′ UTRs), and transcription termination sites (TTSs), were collected and blasted (Figure 2B).

Interestingly, barley enhancers covered intergenic regions (72.9%) much more frequently than intronic regions (3.3%) and CDS regions (6.2%) (Figure 2B), and the distribution was quite different from that in the genomes of *Drosophila*,* Arabidopsis*, and rice, which were smaller and less repetitive. In *Drosophila*, over 55% of the identified enhancers were located within introns, especially in the first intron (∼ 37.2%) [21]. In rice, less than 25% of enhancers mapped by STARR-seq were overrepresented in intergenic regions [27]. In *Arabidopsis*, 73.2% of STARR-seq enhancers were enriched in CDS regions, whereas only 0.2% of the enhancers were enriched in intergenic regions [34]. However, in highly repetitive maize genomes, ∼ 70% of all mapped micrococcal nuclease hypersensitive site (MNase HS) sequences occurred within the intergenic space, despite different identification methods [35]. Thus, the distribution of STARR-seq enhancers between intergenic and non-intergenic regions might be influenced by the genome size, transposable element (TE) composition, and gene density, regardless of the species.

Moreover, for genes overlapped by barley enhancers, the 5′ UTRs (∼ 3.3%) and TSS regions (∼ 6.8%) were overlapped more than the 3′ UTRs (∼ 1.9%) and TTS regions (∼ 5.6%) (Figure 2C). Although the genome size, TE composition, and intergenic spans of barley were quite different from those of rice and *Arabidopsis*, the enhancer distribution trend of 5′ UTR > 3′ UTR was consistent, thus revealing a common *cis*-regulation pattern in plants that enhancers prefer to locate at the 5′ end of genes [27,34]. This result also fits well with our universal understanding that enhancers always act in conjunction with promoter regions.

### Barley enhancers were highly repetitive in sequence

**Figure 3:**
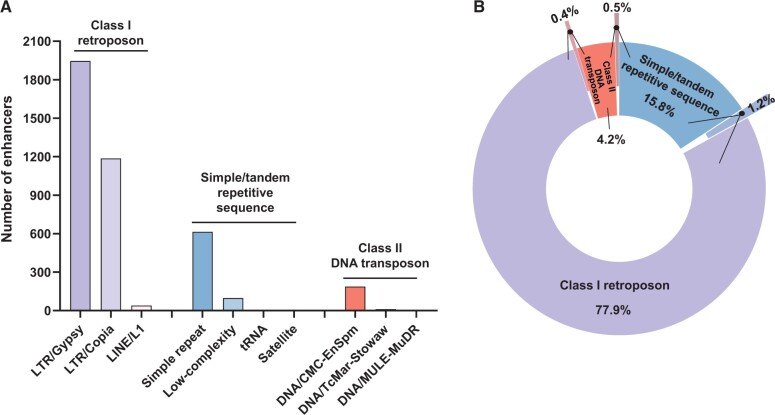
Sequence characteristics of barley STARR-seq enhancers **A**. Number of enhancers in categories of various repetitive sequences. **B**. Proportions of enhancers in categories of class I retroposon, class II DNA transposon, and simple/tandem repetitive sequence, respectively. LTR, long terminal repeated; LINE, long interspersed nuclear element; MULE, Mu-like element; tRNA, transfer RNA; Stowaw, a variant of miniature inverted repeat transposable element.

The enhancers identified in this study were aligned to the MorexV2.44_pseudomolecules_assembly genome, which revealed highly repetitive sequences. RepeatMasker identified 3917 enhancers (∼ 53.5%) with typical repetitive sequences of barley. These enhancers were further divided into three categories: class I retroposons, class II DNA transposons, and simple/tandem repetitive sequences, with approximately 2% of the enhancers involved in more than one repetitive category (**Figure 3**). Class I retroposons accounted for the highest percentage (∼ 79.5%), and class II DNA transposons accounted for the lowest percentage (∼ 5.1%) (Figure 3B). The results revealed that a surprisingly high proportion (84.2%) of STARR-seq enhancers in barley partially overlapped with TEs. In the rice genome, which presented a composition of ∼ 35% TEs, and 52.1% of the identified STARR-seq enhancers were located in the TE regions [27,36]. Obviously, STARR-seq enhancers within the TE regions of both rice and barley accounted for the majority, although the proportion seemed positively correlated with the genomic TE composition. Interestingly, in the maize genome, which was composed of 85% TEs, 4590 out of 32,421 (∼ 14.2%) ATAC-seq-identified ACRs presented at least a partial overlap with annotated TEs [37]. Clearly, TEs generate enhancers to increase gene expression in cereal crops, although the proportion of TEs and enhancers is largely influenced by the sequencing method. Moreover, the results in cereal crops were quite different from that of *Drosophila* enhancers, which were significantly underrepresented in repetitive sequences [21,27].

More specifically, among the highly repetitive enhancers, 1946, 1186, and 39 enhancers were identified as long terminal repeat (LTR)/Gypsy, LTR/Copia, and long interspersed nuclear element (LINE)/L1 enhancers in the class I retroposon category, respectively; 614, 97, 3, and 1 enhancers were recognized as simple repeat, low-complexity, satellite, and transfer RNA (tRNA) enhancers in the simple/tandem repetitive sequence category, respectively; and 187, 10, and 2 enhancers were regarded as DNA/CMC-EnSpm, DNA/TcMar-Stowaw, and DNA/Mutator-like element (MULE)-MuDR enhancers among the class II DNA transposons, respectively (Figure 3A; Table S2). Analysis of the sequence signatures of highly repetitive enhancers further revealed that LTRs, especially LTR/Gypsy, were significantly related to gene expression activities in barley, which is consistent with the fact that nearly 50% of barley TEs are LTR/Gypsy enhancers [38]. In maize, the LTR/Gypsy families were the most significantly enriched enhancer candidates [39]. In rice, LTR-retrotransposons could harbor regions with strong enhancer activities, and some rice STARR-seq enhancers indeed overlapped with LTRs, although the proportion was not clear [27,40]. TEs and LTRs are important components of plant enhancers, although in *Drosophila*, a similar sequence pattern was not observed.

### Barley enhancers may affect genes within 100 kb

**Figure 4:**
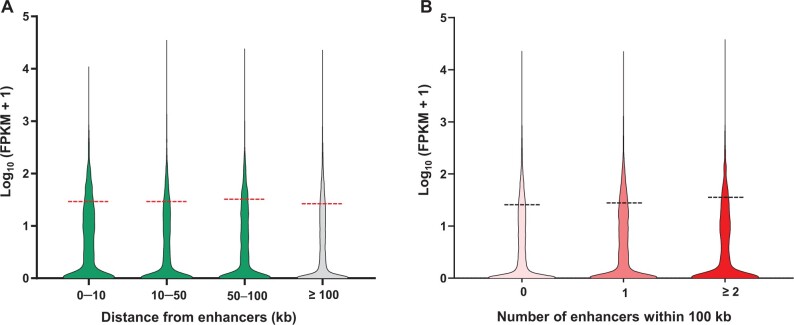
Functional range and enhancing effect of barley STARR-seq enhancers **A**. Overall expression levels of genes within 0–10, 10–50, 50–100, and ≥ 100 kb of barley STARR-seq enhancers. **B**. Overall expression levels of genes having 0, 1, and ≥ 2 enhancers within 100 kb. FPKM, fragments per kilobase million.

In the rice genome, 28.7% of genes have at least one proximal enhancer (gene body ± 5 kb), with the majority of STARR-seq enhancers mapped within or close to genes [27]. In the larger maize genome, only 32.5% of ACRs occur at > 2 kb from their nearest genes and only 12.7% of enhancers occur at > 20 kb [28]. However, in the barley genome with significantly lower gene density, the conclusion is different. Few genes had enhancers within the traditional ±10 kb distance (∼ 5.8%). To roughly assess the functional range of barley enhancers, abundant RNA sequencing (RNA-seq) data of barley genes were collected from BioProject (BioProject: PRJNA431836, PRJNA495764, PRJNA496380, PRJNA558196, PRJEB39864, PRJEB34186, and PRJNA639036) and Gene Expression Omnibus (GEO: GSE167271) in the National Center for Biotechnology Information (NCBI), and then fragments per kilobase million (FPKM) values were calculated (Table S3). The overall expression levels of genes within 0–10, 10–50, 50–100, and ≥ 100 kb of STARR-seq enhancers indicated that genes located within 100 kb of barley enhancers presented higher expression relative to those located greater than 100 kb away, while the overall expression levels of genes within 0–10, 10–50, and 50–100 kb were similar (**Figure 4**A). This result suggests the possibility that barley enhancers influence gene expression within a broader range of 100 kb. A large proportion of repetitive sequences and greater distances between genes in barley possibly blocked the enhancers from regulating nearby genes. Compared to rice (approximately 125 genes/Mb) [36] and maize (approximately 20 genes/Mb) [41], the lower gene density in barley (approximately 5 genes/Mb) [42] might have driven enhancers to adapt to a much broader functional range.

To further validate this hypothesis, quantitative analyses of barley genes with or without enhancers in a 100-kb range were performed (Table S4). As expected, despite not reaching a significant level, the overall expression level of the 12,129 barley genes with enhancers within 100 kb was higher than that of the 30,701 barley genes without enhancers within 100 kb (average FPKM value: 28.95 > 26.50). More interestingly, the overall expression level of genes with ≥ 2 enhancers within 100 kb was improved compared with those with only one enhancer (average FPKM value: 32.37 > 28.95), thus revealing potential accumulative effects of barley enhancers (Figure 4B). In general, the enhancers we identified worked as up-regulatory elements in barley at the omics level.

### Enhancers of different repeat categories played different roles in gene expression

**Figure 5:**
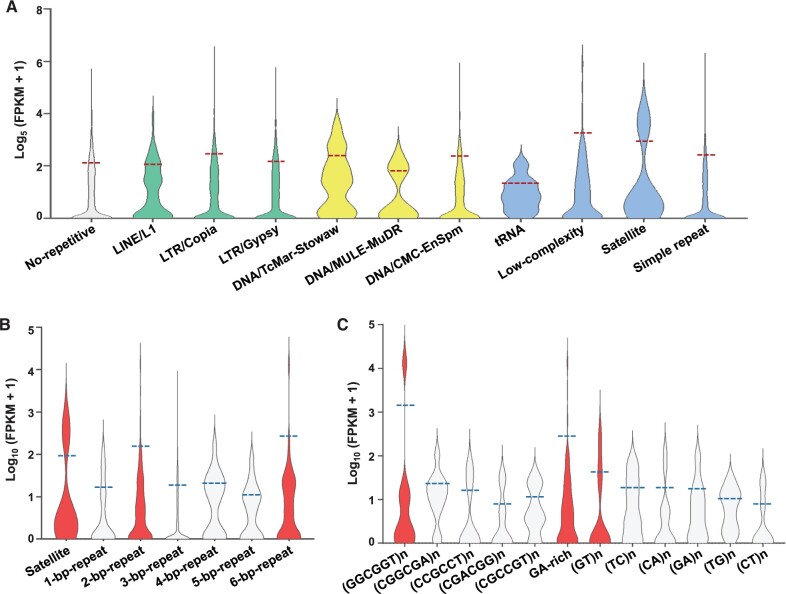
Enhancing effect analysis of barley STARR-seq enhancers in different categories of repetitive sequence **A**. Overall expression levels of genes having no-repetitive sequence enhancers or having 10 categories of repetitive sequence enhancers. **B**. Overall expression levels of genes within 100 kb of enhancers in the simple/tandem repetitive sequence category with the groups divided by base pair number. **C**. Overall expression levels of genes within 100 kb of enhancers in 2-bp-repeat and 6-bp-repeat groups shown in (B); and the groups were divided by base pair composition of enhancers.

Although class I retroposon TEs, namely, LTR/Copia and LTR/Gypsy enhancers, were identified as the main types of repetitive barley enhancers, their enhancing effects might not be the best (**Figure 5**A; Table S5). After separately analyzing the average expression levels of genes within 100 kb of enhancers in the no-repetitive sequence category and 10 repetitive sequence categories (LINE/L1, LTR/Copia, LTR/Gypsy, DNA/TcMar-Stowaw, DNA/MULE-MuDR, DNA/CMC-EnSpm, tRNA, low-complexity, satellite, and simple repeat enhancers), enhancing effects were preliminarily assessed. Surprisingly, satellite, simple repeat, and low-complexity enhancers with the simplest tandem repeat sequences exhibited the best enhancing effects (Figure 5A; Table S5). Retroposon-related enhancers (LINE/L1, LTR/Copia, and LTR/Gypsy) could be regarded as tracing marks in the largest proportion, although their enhancing effects were not very strong (Figure 5A). Enhancers without repetitive sequences tended to play the weakest regulatory roles, thus revealing the importance of redundant repeats in the *cis*-regulation of gene expression in barley (Figure 5A).

Moreover, more specific repetitive types of the best-performing satellite, simple repeat, and low-complexity enhancers were analyzed in detail. According to the number of base pairs, satellite, 2-bp-repeat, and 6-bp-repeat enhancers were the strongest types (Figure 5B; Table S6). Among all base compositions, GA-rich, (GT)n, (GGCGGT)n, or satellite enhancers had the most positive effects on gene expression levels within 100 kb at the genomic level (Figure 5C; Table S6).

### Barley STARR-seq enhancers were successfully validated by a tobacco system

**Figure 6:**
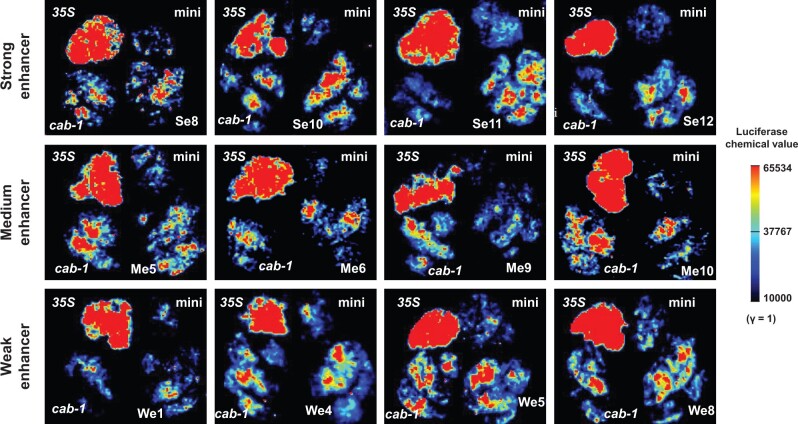
Chemical luciferase imaging of representative strong, medium, and weak enhancers *35S* represents the firefly *Luc* gene driven by the CaMV *35S* promoter only (a positive control); mini represents the firefly *Luc* gene driven by the minimal CaMV *35S* promoter only (a negative control); *cab-1* represnts the firefly *Lu*c gene driven by the minimal CaMV *35S* promoter together with an upstream *cab-1* enhancer (a positive control); Se, Me, and We represent the firefly *Luc* gene driven by the minimal CaMV *35S* promoter together with upstream strong, medium, and weak enhancers, respectively. Scale bar of luciferase chemical values referred to the “Image Lab” instrument parameter (γ = 1). *Luc*, luciferase.

A total of 45 peaks were successfully cloned into the pGreen II-0800-*Luc* (empty) vector carrying the minimal CaMV *35S* promoter. Using the empty vector with both the minimal CaMV *35S* promoter and the *cab-1* enhancer [31] and the empty vector with the CaMV *35S* promoter as two positive controls as well as the empty vector with the minimal CaMV *35S* promoter as the negative control, the dual-luciferase reporter assay system revealed that the majority of our enhancers were effective in promoting luciferase (reporter) expression via preliminary imaging. Randomly chosen enhancers with strong, medium, and weak enrichment values showed an obvious enhancing effects compared with the minimal CaMV *35S* promoter only; however, the degrees were quite different. The partial chemi-imaging results are shown in **Figure 6**.

**Figure 7:**
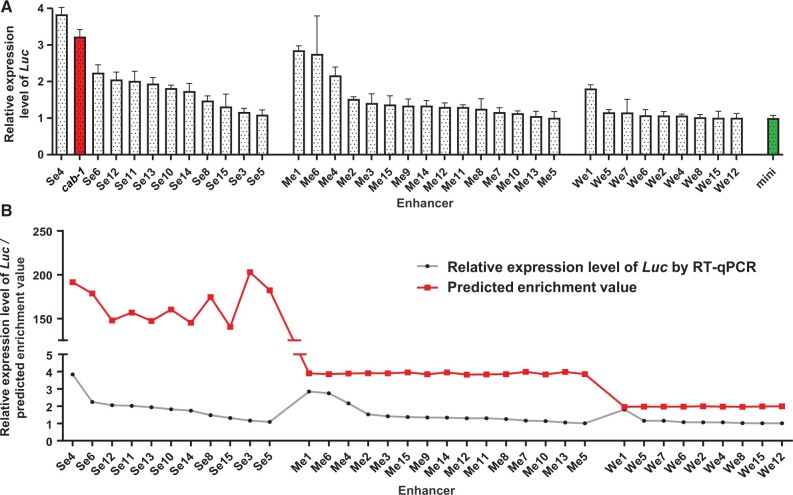
RT-qPCR validation of 35 effective STARR-seq enhancers **A**. Relative expression levels of *Luc* enhanced by 35 effective STARR-seq enhancers. 35 enhancers were included in the 45 randomly chosen enhancers from strong, medium, and weak categories. Expression level of *Renilla Luc* was used as an internal control. Column in red represents the relative expression level of *Luc* up-regulated by the *cab-1* enhancer (a positive control); column in green represents the relative expression level of *Luc* promoted by minimal CaMV *35S* promoter only and was normalized to 1 (a negative control). **B**. Fitted curves between predicted enrichment values and RT-qPCR relative expression levels of *Luc* enhanced by 35 effective enhancers. RT-qPCR, quantitative reverse transcription polymerase chain reaction.

To further quantify the effects of enhancers predicted by STARR-seq, quantitative reverse transcription polymerase chain reaction (RT-qPCR) was performed on the 45 enhancer treatments. As expected, 35 of 45 (∼ 77.8%) enhancer sequences were successfully validated, with 11, 15, and 9 sites having strong, medium, and weak strengths, respectively. The average enhanced levels were quantified by the enhancer/mini values (the expression level of *Luc* driven by the minimal CaMV *35S* promoter without enhancer was normalized to 1). The predicted strong enhancer sites increased the expression levels of *Luc* by an average of 1.885 times, which was higher than that of the medium sites (∼ 1.534 times) and weak sites (∼ 1.154 times) (**Figure 7**A). Notably, enhancer Se4 in the strong catalog even exceeded the effect of the reported positive control *cab-1*. Our results showed that the effects of enhancers in plants were highly correlated with our STARR-seq enrichment values (Figure 7B).

In conclusion, the enrichment levels of candidate enhancers were significantly associated with the up-regulated levels of luciferase. Both the enhancer sequences and enhancing strengths predicted from our STARR-seq data were reliable for further functional analysis and utilization.

### H3K4me3 and H3K27me3 were enriched at barley STARR-seq enhancers

**Figure 8:**
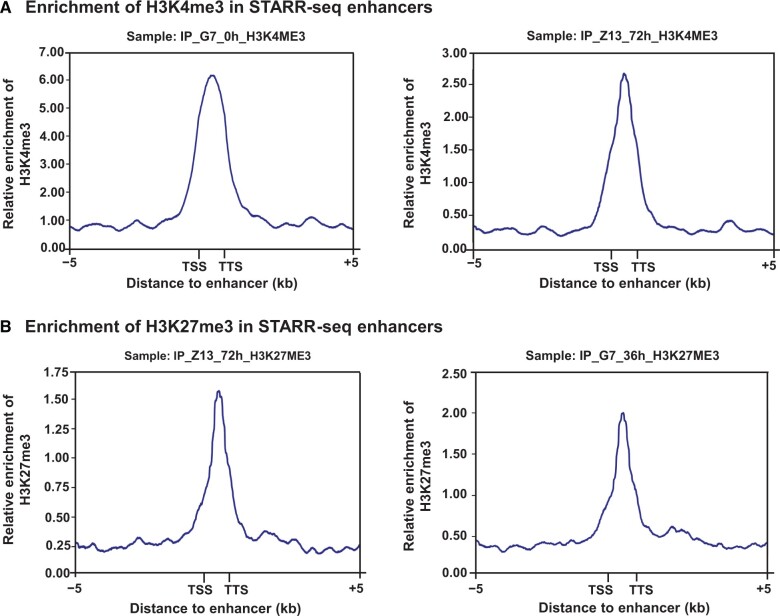
Histone modification marks enriched in barley STARR-seq enhancers Enrichment of H3K4me3 (**A**) and H3K27me3 (**B**) marks in the barley STARR-seq enhancers. ChIP-seq data of different samples were downloaded from the NCBI Gene Expression Omnibus (GEO: GSE122539), with H3K4me3 and H3K27me3 marks within ±5 kb of STARR-seq enhancers. The distance from TSS to TTS represents the enhancer body; and the blue peaks represent the enrichment. ChIP-seq, chromatin immunoprecipitation sequencing.

Histone modification marks of barley STARR-seq enhancers were analyzed by collecting published ChIP-seq data (GEO: GSE122539). The active mark H3K4me3 and repressive mark H3K27me3 were included in the analysis [43,44]. In a previous study, H3K4me3 and H3K27me3 were both enriched in rice STARR-seq enhancers [27]. A similar result was observed for histone modifications in barley. Both the active mark H3K4me3 and repressive mark H3K27me3 were enriched in our barley STARR-seq enhancers, with obvious peaks (**Figure 8**). Interestingly, H3K27me3 has been reported to restrict or inhibit enhancer activity [45–47]. Anomalous H3K27me3 enrichment, both in rice and barley, further confirmed that H3K27me3 is mostly associated with a repressed chromatin state in animals, although in plant genomes, it may represent a dependent enhancer mark regardless of whether it is latent or active.

Throughout the genomes of mice, *Drosophila*, humans, rice, maize, and wheat, H3K27ac, H3K9ac, and H3K36me3 are conserved enhancer marks, while H3K4me1 is underrepresented in rice, maize, and wheat but actively enriched in *Drosophila* and humans [15,21,27,39,48,49]. Unfortunately, ChIP-seq data have not been reported on H3K27ac, H3K9ac, H3K36me3, or H3K4me1 in barley.

## Discussion

### Barley enhancers identified by STARR-seq are highly reliable

The expression levels of barley mRNAs with or without STARR-seq enhancers within 100 kb revealed the authenticity and reliability of the 7323 predicted enhancers at the omics level, and mRNAs with STARR-seq enhancers within 100 kb tended to exhibit significantly higher expression. Moreover, the dual-luciferase reporter assay system experimentally illustrated the reliability of our predicted enhancers, with over 70% of the candidates validated in heterologous systems. However, because certain tissue-specific enhancers are stimulated by light or time, the system itself cannot fully represent the potential of our enhancers with hidden functions [29,50]. The remaining enhancers that do not work in tobacco may also work well in barley. Imaging and RT-qPCR results in tobacco revealed the authenticity and reliability of 7323 predicted enhancers at the experimental level.

### Commonalities and characteristics of barley enhancers

The location signature of barley enhancers was further investigated in this study. STARR-seq enhancers identified in the *Drosophila*, *Arabidopsis*, and rice genomes were mostly located close to or within genes [21,27,34]. Among the maize and barley enhancers, the majority were located in intergenic regions, which was partially because of the different genomic compositions [35]. Interestingly, compared with the 3′ UTRs and TTS ± 50 bp regions, the 5′ UTRs and TSS ± 50 bp regions had a relatively higher proportion of barley, rice, and *Arabidopsis* STARR-seq enhancers [27,34]. The results mirrored the special location preference of plant enhancers. The activity of enhancers is always lower when located downstream of the gene than upstream of the minimal *35S* promoter [29]. The location distribution feature may help maximally utilize plant enhancers.

Meanwhile, the sequence signatures of plant enhancers tend to be highly repetitive in the grass family, with the majority of enhancers existing as TEs [27,37,51–53]. Despite their commonality, barley STARR-seq enhancers also have unique sequence features. In barley, a significantly greater number of retroposon-related enhancers (∼ 79.5%), especially LTR/Gypsy enhancers, was observed relative to DNA transposon-related enhancers (5.1%). An evolutionary analysis among Triticeae crops indicates that the LTR/Gypsy element is one of the main reasons for genome expansion [38]. High-proportion LTR/Gypsy enhancers might play a vital role in both gene expression and evolution, and retroposons could represent an available label for tracing enhancers in both animals and plants [54,55].

A broader functional range of barley enhancers has also been proposed. Traditionally, 10 kb or less has been regarded as the strengthening range of enhancers in both mammals and plants with small genomes, such as rice and *Arabidopsis* [6,55–58]. However, barley enhancers have the potential to significantly broaden the range to 100 kb via gene expression analysis. As reported in maize, the long-distance interaction enhancers of genes *booster1* (*b1*), *teosinte branched1* (*tb1*), *vegetative to generative transition 1* (*Vgt1*), and *bx1* (*benzoxazinless1*) could *cis-*regulate target genes at distances of approximately 100, 70, 60, and 140 kb, respectively [1,3,59–61]. The high proportion of TEs and large intergenic spaces in barley not only generate enhancers but might also promote their regulation of additional genes.

### Repetitive enhancers have huge potential for gene regulation

The role of repetitive sequences is further emphasized in this study, and LTRs have long been reported to enhance human responses [62–64]. In maize, an 853-bp tandem repeat sequence located 100 kb upstream of *b1* regulates *b1* expression [59,65]. A miniature inverted-repeat TE inserted 70 kb upstream of the maize AP2/ERF transcription factor coding gene (*ZmRap2.7*) could regulate *ZmRAP2.7* expression by affecting epigenetic modification [60]. In this study, the expression analysis indicates that enhancers of simple/tandem repetitive sequences have the best enhancing activity, thereby partially contradicting the previous hypothesis that tandem repeats act as weak enhancer silencers to modulate the expression of proximal genes [66]. Our study further emphasizes the importance of enhancers in repetitive sequences and indicates that they might strongly enhance the expression levels of important functional genes to adapt to a transient environment.

In addition, three dominant repeat types of barley enhancers, GA-rich, (GT)n, and (GGCGGT)n, were identified. Tandemly repeated GA-rich sequences have been frequently reported as motifs within highly active promoter regions and thus show good potential as strong enhancers in animals and humans [67–69]. A (GT)n polymorphism in the promoter/enhancer region of forkhead box protein P3 (*FOXP3*) is associated with the development of severe acute graft-versus-host disease (GVHD) in humans [70]. Although such effects have not been reported in plants, in our study, the RNA-seq data analysis results partially support the enhancing effects of “GA-rich” and “(GT)n” enhancers. These two enhancers may be common and conserved sequence signatures of the active enhancers. In addition, the newly discovered (GGCGGT)n enhancer may be another sequence signature for tracing highly active plant enhancers.

## Conclusion

In summary, we identified 7323 enhancers in barley and validated their authenticity via RNA-seq data analysis and experimental RT-qPCR quantification. Intergenic and repetitive barley enhancers were found in high proportions, and the marks H3K4me3 and H3K27me3 were enriched. LTR enhancers were the most enriched, while simple tandem repetitive enhancers showed the greatest enhancement, thus demonstrating enhancer characteristics unique to barley.

## Materials and methods

### Reporter plasmid construction and library preparation

To comprehensively identify sequences with enhancer activity in the barley cultivar Morex, we constructed reporter and plasmid libraries from randomly ultrasonicated genomic DNA fregments ranging from 500 bp to 800 bp. The input plasmid DNA library was separately transfected into protoplasts multiple times (4–6 times) to increase the abundance, and protoplasts were transfected and then randomly divided into two replicates for library construction.

The library preparation protocols mainly referred to a previous study on rice with the same modified plasmid pBI221 [27]. First, 1 ml genomic DNA (> 100 ng/μl) was obtained from two-week-old barley seedlings. Genomic DNA was then fragmented by non-contact sonication (20% power at 5 s on and 10 s off, which was repeated 14 times in a liquid volume of 1 ml). DNA fragments ranging from 500 bp to 800 bp were separated by 1% agarose electrophoresis and maximally collected using a FastPure Gel DNA Extraction Mini Kit (Catalog No. DC301-01, Vazyme Biotech, Nanjing, China). The purified DNA products were repaired and ligated to VAHTS DNA Adapters Set1/Set2 for Illumina (Catalog No. N801/802-01/02, Vazyme Biotech). The ClonExprress II One Step Cloning Kit (Catalog No. C112, Vazyme Biotech) was used to successfully ligate the adaptor-ligated genomic DNA into the pBI221 vector, which was linearized by the incision enzymes *Bsr*GI and *Mlu*I. Different ratios (> 10) of DNA fragments/linearized pBI221 vectors were generated to avoid connection preference of short fragments. Ligation products were transformed into DH5α strains (Catalog No. DL1001, Weidi Biotechnology, Shanghai, China) and cultured at 37°C for 12–16 h. The cultivation volumes were further increased to 5–6 l using Luria–Bertani medium, and the reporter plasmids were purified using the E.Z.N.A. Endo-Free Plasmid Maxi Kit (Catalog No. D6926, Omega Bio-tek, Norcross, GA). The bacterial endotoxin of the input plasmid was removed by adding 5 mol/l NaCl, a mixture solution of 30% polyethylene glycol 6000 (PEG 6000) and 1.5 mol/l NaCl, and 70% ethanol in turn. The purified plasmid was collected and quantified using a NanoDrop One (Thermo Fisher Scientific, Waltham, MA).

### Protoplast transfection

Protoplasts were isolated from 20–30 etiolated leaves of barley seedling leaves after 2 weeks of cultivation. Etiolated seedlings were chopped into strips of 0.5–1 mm and digested with Cellulase R-10 (Catalog No. 9012-54-8, Beijing Lablead Biotech, Beijing, China) and Macerozyme R-10 (Catalog No. 200115-03, Yakult Pharmaceutical, Tokyo, Japan). The cell walls were digested for ∼ 4 h by shaking at 45–50 r/min under dark conditions at 25°C in multi-amplitude rail shaker (Catalog No. ZWY-100H, ZHICHENG, Shanghai, China). A 200 nylon mesh filter was used to sieve the enzymatic hydrolysate, and W5 solution (154 mM NaCl, 125 mM CaCl_2_, 5 mM KCl, and 2 mM 4-morpholineethanesulfonic acid, pH 5.7) was used to repeatedly wash the protoplasts. Protoplasts were collected by centrifugation at 100 *g* at 4°C. For transfection, 40 μg of reporter plasmid DNA (≥ 2 μg/μl) was gently blended with 200 μl of protoplasts (approximately 1 × 10^6^ cells) in a non-stick centrifuge tube, and then 220 μl of freshly prepared PEG 4000 solution (40%, w/v) (Catalog No. 807490, Sigma-Aldrich Biotech, St. Louis, MO) was added to mediate transfection in the dark. The W5 solution was used again to wash off the 40% PEG 4000 and terminate the reaction after 15–20 min. Finally, the protoplasts were incubated in W5 solution at 25°C for 16 h under dark conditions.

### Illumina sequencing library construction for reporter cDNA and input plasmid libraries

A VAHTS Universal DNA Library Prep Kit for Illumina V3 (Catalog No. ND607, Vazyme Biotech) and VAHTS mRNA-seq V2 Library Prep Kit for Illumina (Catalog No. NR601, Vazyme Biotech) were used to construct input plasmid libraries and reporter cDNA libraries, respectively, using nearly the same flow path. Specifically, both mRNA and plasmid DNA from the transfected protoplasts were extracted using a Plant RNA Collection Kit v1.5 (Catalog No. RN33050, Biofit, Chengdu, China) without DNA elimination. VAHTS mRNA Capture Beads (Catalog No. N401, Vazyme Biotech) were used to capture RNA only, and VAHTS DNA Clean Beads (Catalog No. N411, Vazyme Biotech) were used to capture DNA from the remaining solution. cDNA was obtained using PrimeScript RT reagent Kit [Catalog No. RR047A, Takara Biomedical Technology (Beijing), Beijing, China] using a DNA elimination process.

Next, nested PCR was performed to amplify the cDNA and DNA using Phanta Max Super-Fidelity DNA Polymerase (Catalog No. P505, Vazyme Biotech) for less than 25 cycles in total. PCR products from the first round were purified using VAHTS DNA Clean Beads (0.8×) and then used as templates for the second round of PCR amplification with primers which included the sequences of VAHTS DNA Adapters. VAHTS DNA Clean Beads (0.8×) were used again for purification. The total PCR cycles of the cDNA and DNA libraries should be the same and as few as possible. Both libraries were sequenced on a NovaSeq PE150 platform at Berry Genomics (Beijing, China).

### Identification of barley STARR-seq enhancers

The identification procedure for STARR-seq data was based on Sun et al. [27] with some adjustments. First, the raw reads of the two replicates were merged to maximize the STARR-seq enhancers. Bowtie2 was used to map the sequence data to the *Hordeum vulgare* genome (MorexV2.44_pseudomolecules_assembly; http://plants.ensembl.org/Hordeum_vulgare/Info/Index) [71,72]. SAMtools was used to filter the mapped reads and store read alignments against the reference sequences [73]. The R package was used for STARR-seq enhancer identification [21]. BasicSTARRseq [74] and Bonferroni correction [75] were performed to adjust the *P* values. The “deduplicate” parameter in BasicSTARRseq was utilized to eliminate redundant data caused by PCR amplification. The genomic region was identified as an enhancer “peak” if the enrichment of cDNA over the input plasmid insert was > 1.3 fold and the adjusted *P* value was < 0.001.

### Location and sequence characteristic analyses of identified enhancers

Chromosomal information, location information, and gene annotation files were downloaded from the MorexV2.44_pseudo-molecules_assembly reference genome of Ensembl Plants (http://plants.ensembl.org/index.html). Based on the aforementioned information, the locations of all identified STARR-seq enhancers were subjected to an orderly Blast search with various gene elements to obtain position relationships and preferences. The distribution of enhancers across the barley genome was depicted by the Gene Location Visualize function in TBtools [76]. RepeatMasker was used to blast 7323 enhancers with known repetitive types in barley [77], and detailed repetitive sequence names and categories were output directly.

### Comparison between STARR-seq enhancers and ATAC-seq enhancers

With the help of the “Gene Location Visualize” function in TBtools, the chromosomal distribution and density characteristics of the STARR-seq enhancers in rice and barley and ATAC-seq peaks in rice, barley, and maize were all determined [27,33,76]. To further cross-compare our STARR-seq enhancers, raw ATAC-seq data of barley and rice were collected from a previous study and downloaded from the Sequence Read Archive in the NCBI (SRA: SRR8742457, SRR8742458, SRR8742459, SRR8742447, SRR8742448, and SRR8742449) [33]. ATAC-seq data were aligned to the same reference genome and analyzed using the same parameters as the STARR-seq data, and the identified ACRs whose “summits” with the highest Tn5 integration frequency fell into the peak ranges of the STARR-seq were considered “overlapped”.

### Expression analysis of enhancers and genes in barley

Approximately 42,831 genes (∼ 5,100,000,000 bp) were annotated in the whole genome of barley, indicating that the average distribution of genes in the barley genome is ∼ 1 gene/100 kb. To assess whether the functional range of barley enhancers is within 100 kb, the overall expression levels of genes within 0–10, 10–50, 50–100, and ≥ 100 kb of STARR-seq enhancers were calculated. The RNA-seq data of 228 samples that were randomly related to abiotic resistance, disease resistance, and growth and development were collected from published online databases from BioProject (BioProject: PRJNA431836, PRJNA495764, PRJNA496380, PRJNA558196, PRJEB39864, PRJEB34186, and PRJNA639036) and Gene Expression Omnibus (GEO: GSE167271) in the NCBI [77–85]. RSEM software was used to quantify all genes, and the FPKM values were acquired [86]. To further check the 100-kb functional range, FPKM values of genes without enhancers within 100 kb, genes with one enhancer within 100 kb, and genes with ≥ 2 enhancers within 100 kb were further calculated.

To evaluate the overall enhancing effects of enhancers with different repetitive types, 7323 enhancers were divided into 11 categories (1 category of no repeats and 10 categories of repetitive sequences). The FPKM values of genes within 100 kb of the 11 categories of enhancers were calculated. The genes of each category, which had obviously high expression levels within 100 kb of enhancers, were further divided by the base pair number and composition of enhancers.

### Experimental validation by dual-luciferase reporter assay system

The 7323 identified enhancers were divided into strong (> 4-fold enrichment), medium (2-to-4-fold enrichment), and weak (< 2-fold enrichment) groups according to their predicted enrichment values [27]. The top 100 enhancers in each group were chosen, of which 15 STARR-seq sites were randomly selected from each group (45 in total). A dual-luciferase reporter assay was performed on the lower epidermis of tobacco leaves for each enhancer. To fully demonstrate the effects of the enhancers, we modified the original pGreenII0800-*Luc* vector by adding a minimal CaMV *35S* promoter before the firefly *Luc* gene. The modified pGreenII0800-*Luc* was linearized using *Kpn*I, and 45 enhancer sequences were separately ligated into the cutting site before the minimal CaMV *35S* promoter. The PCR primers are listed in Table S7.

A CaMV *35S* promoter was added before the firefly *Luc* gene in the original pGreenII0800-*Luc* to generate a positive control, and it showed strong luciferase fluorescence. The empty modified pGreenII0800-*Luc* was used as a negative control, and it showed almost no luciferase fluorescence. Moreover, the previously reported wheat enhancer *cab-1* was ligated into the *Kpn*I site of the modified pGreenII0800-*Luc* as another positive control. After *Agrobacterium* transformation and positive detection, the three control groups were synchronously injected with different parts of the same *N. tabacum* L. leaves, with one STARR-seq enhancer waiting for verification. After 36–48 h of cultivation, D-luciferin was uniformly smeared on the lower epidermis and luciferase fluorescence was imaged using ChemiDoc MP Imaging System [Bio-Rad Laboratories (Shanghai), Shanghai, China]. The RNA of the tobacco leaves under different treatments was separately extracted using the Plant RNA Collection Kit v1.5 (Catalog No. RN33050, Biofit). RT-qPCR for the firefly *Luc* gene was performed to quantify the effect of each predicted enhancer. The *Renilla Luc* gene was used as an internal control.

### Histone modification analysis based on available ChIP-seq data

To reveal the histone modification levels of barley STARR-seq enhancers, ChIP-seq data for barley were downloaded from the Gene Expression Omnibus in the NCBI (GEO: GSE122539) [44], including data on H3K27me3 and H3K4me3. MACS2 was used to call the peaks of the obtained histone modification data [87], and Bowtie 2 was used to align the STARR-seq data of both input plasmid libraries and cDNA libraries to the MorexV2.44_pseudomolecules_assembly genome [88]. deepTools was then utilized to count the number of ChIP-seq reads overlapped in a ±5 kb range from the STARR-seq enhancers, and plot profiles were configured by the “computeMatrix” function in deepTools [89].

## Supplementary Material

qzae012_Supplementary_Data

## Data Availability

The raw STARR-seq data in this study have been deposited in the Genome Sequence Archive [90] at the National Genomics Data Center, Beijing Institute of Genomics, Chinese Academy of Sciences / China National Center for Bioinformation (GSA: CRA009174), and are publicly accessible at https://ngdc.cncb.ac.cn/gsa.

## CRediT author statement

**Wanlin Zhou:** Writing – original draft, Validation, Formal analysis. **Haoran Shi:** Software, Formal analysis, Visualization. **Zhiqiang Wang:** Methodology, Investigation. **Yuxin Huang:** Validation, Investigation. **Lin Ni:** Validation, Investigation. **Xudong Chen:** Resources, Data Curation. **Yan Liu:** Writing – review & editing. **Haojie Li:** Validation, Investigation. **Caixia Li:** Writing – review & editing. **Yaxi Liu:** Conceptualization, Supervision, Funding acquisition. All authors have read and approved the final manuscript.
