# Supplementary material for: Identification of Highly Repetitive Enhancers with Long-range Regulation Potential in Barley via STARR-seq
Source: Genomics Proteomics Bioinformatics. 2024 Feb 21;22(2):qzae012. doi: 10.1093/gpbjnl/qzae012 (PMC12016029; doi:10.1093/gpbjnl/qzae012)
Supplement: qzae012_Supplementary_Data [file qzae012_supplementary_data.zip › qzae012_Supplementary_Data/Supplementary material captions.docx]

**Supplementary material**

**Figure S1 Chromosomal distribution of barley ATAC-seq peaks**

Chromosomal length was labeled on the left and the scale bar was on the right. Scale values of the corresponding color represented the number of enhancers per Mb (enhancer density). ATAC-seq, assay for transposase-accessible chromatin using sequencing.

**Figure S2 Comparison between rice ATAC-seq and STARR-seq peaks**

**A.** Chromosomal distribution of rice STARR-seq peaks. **B.** Chromosomal distribution of rice ATAC-seq peaks. Chromosomal length was labeled on the left and the scale bar was on the right. Scale values of the corresponding color represented the number of enhancers per 100 kb (enhancer density)

**Figure S3 Chromosomal distribution of maize ATAC-seq peaks**

Chromosomal length was labeled on the left and the scale bar was on the right. Scale values of the corresponding color represented the number of enhancers per Mb (enhancer density).

**Table S1 Information of identified STARR-seq enhancers in cultivar Morex**

**Table S2 Information of enhancers in various repeatitive sequence categories via RepeatMasker**

**Table S3 FPKM data of barley genes within different range of STARR-seq enhancers**

**Table S4 FPKM data of barley genes having 0, 1, and** ≥ **2 enhancers within 100 kb**

**Table S5 FPKM data of barley genes within 100 kb of enhancers in 11 different repeat categories**

**Table S6 FPKM data of barley genes within 100 kb of enhancers with different repetitive base pair composition**

**Table S7 Primers used in enhancers’ vector construction**
