# Supplementary figures and images for: Identification of Highly Repetitive Enhancers with Long-range Regulation Potential in Barley via STARR-seq
Source: Genomics Proteomics Bioinformatics. 2024 Feb 21;22(2):qzae012. doi: 10.1093/gpbjnl/qzae012 (PMC12016029; doi:10.1093/gpbjnl/qzae012)

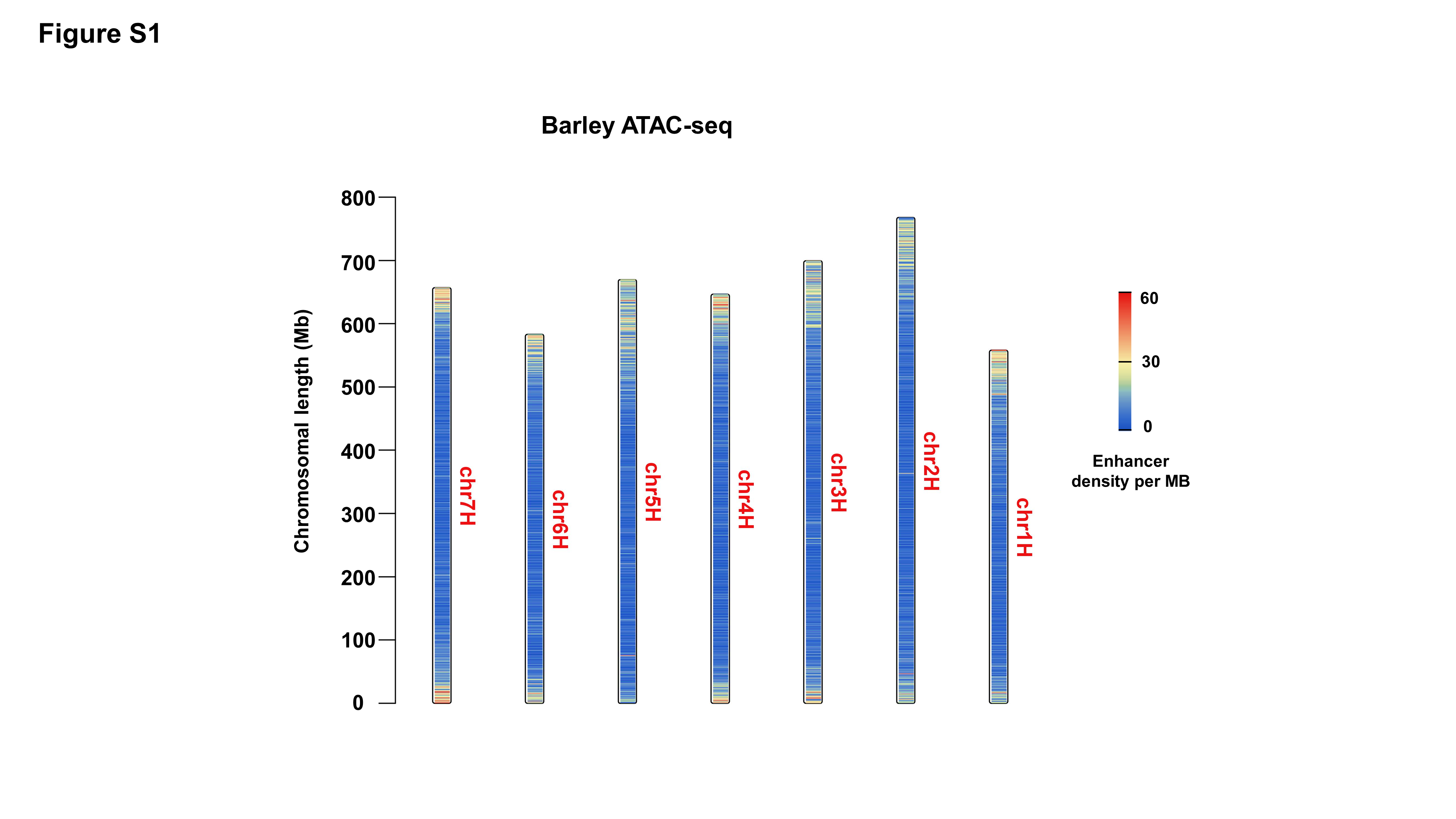

Supplement: qzae012_Supplementary_Data [file qzae012_supplementary_data.zip › qzae012_Supplementary_Data/Figure S1.tif]

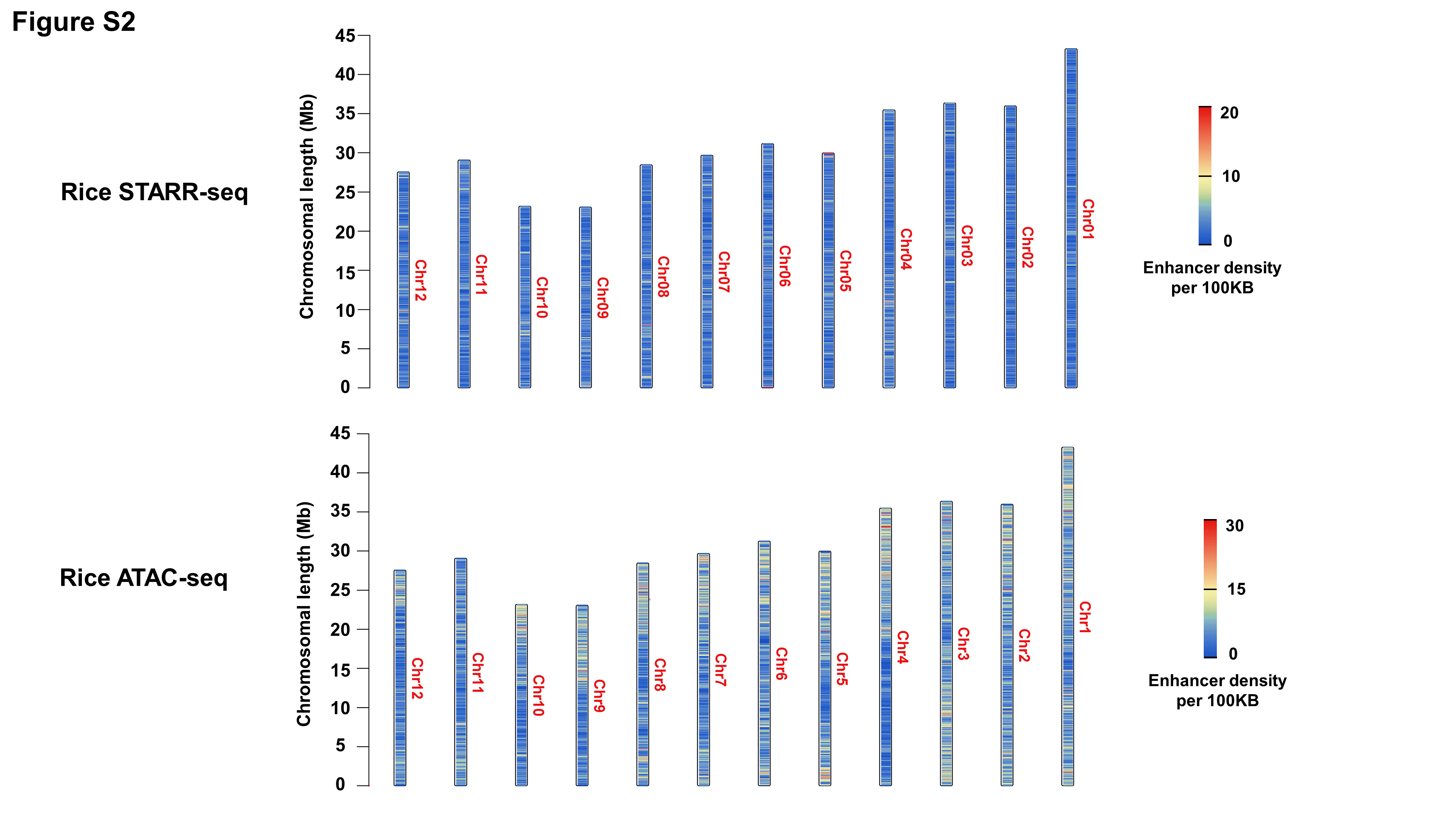

Supplement: qzae012_Supplementary_Data [file qzae012_supplementary_data.zip › qzae012_Supplementary_Data/Figure S2.tif]

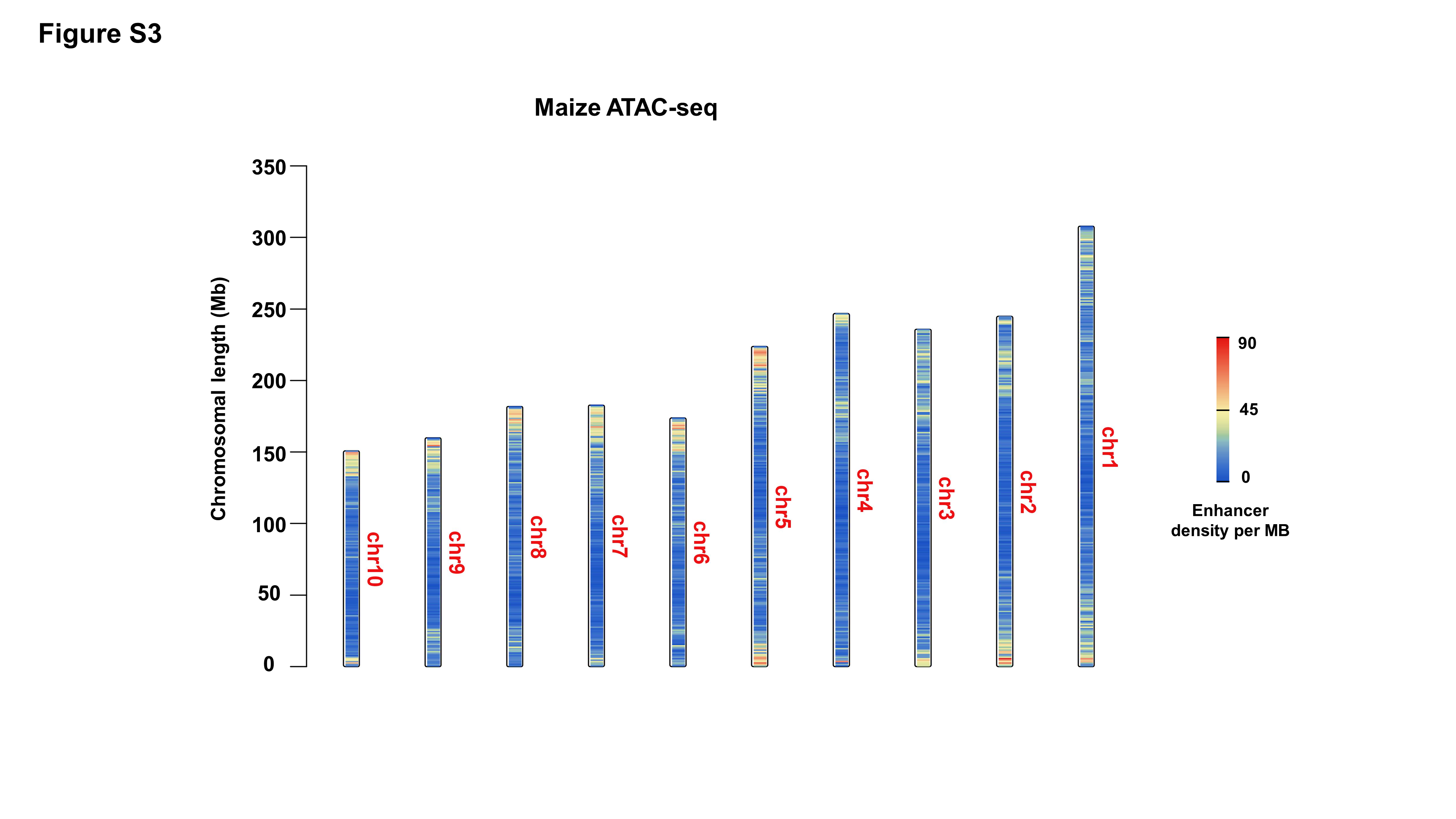

Supplement: qzae012_Supplementary_Data [file qzae012_supplementary_data.zip › qzae012_Supplementary_Data/Figure S3.tif]
